# Lumping disparate emergency dispatch priority levels together creates an assumption error regarding ‘overtriage’

**DOI:** 10.1186/s13049-025-01425-z

**Published:** 2025-07-01

**Authors:** Greg Scott, Jeff Clawson, Christopher Olola, Matthew Miko, Bryon Schultz, Brett Patterson

**Affiliations:** International Academies of Emergency Dispatch, 110 S. Regent Street, Salt Lake City, UT USA

Dear Editor,

As the stewards of the Medical Priority Dispatch System (MPDS^®^)(Priority Dispatch Corp., Salt Lake City, Utah, USA), we would like to respond to the authors of the recent article entitled “Performance Measures of the Medical Priority Dispatch System in an Urban Basic Life Support System”, Nicoletta et al. [1] We commend the investigators for addressing this very important research topic. This study, however, contains a major design flaw that leads to an assumption error regarding ‘overtriage’ within the MPDS.

In its design, the study lumps three disparate dispatch priority levels together, classifying them all as ‘urgent.’ This ignores their proven fundamental differences as well as the fact that many paramedic treatment and transport decisions are independent of the patient’s need as determined at dispatch, in particular for potentially at-risk patients discovered by the Emergency Medical Dispatcher (EMD) who ultimately turn out to be clinically stable after a more thorough and detailed face-to-face evaluation by a trained Emergency Medical Services (EMS) responder. This study incorrectly labels all such cases as ‘overtriage’ at dispatch.

For example, a patient in the known cardiac-risk age range (≥ 35 years) with chest pain, but no other symptoms, based on current standards of care and practice, will require a timely response from an Advanced Life Support (ALS) crew to do a comprehensive cardiac assessment—regardless of the ultimate findings, treatment, and/or transport decision made by the attending paramedic crew. The MPDS determinant code for such a patient would be a 10-C-3 (Breathing normally [≥ 35]). This code reflects the patient’s need for an ALS assessment in a timely manner—which indeed is the standard of care and practice for all state-of-the-art EMS services. Further, this code does not mandate a lights-and-siren response; to the contrary, the MPDS generic response is a single ALS ambulance, cold response (no lights-and-siren). Similarly, the patient’s need and accepted standards of practice extend to higher priority cases triaged by the MPDS. Consider another chest pain patient over age 35, but now with an additional symptom such as not alert, clammy, severe respiratory problems (difficulty speaking between breaths), changing color, or a history of heart attack or angina. In such cases, the chances of the patient having an acute myocardial infarction (AMI) are increased as compared to the previous example, hence one of the 10-D (higher priority DELTA level) codes in the MPDS is assigned (Fig. 1). In this case, the accepted practice is an immediate (lights-and-siren) response from an ALS crew—this is true regardless of whether or not the crew discovers an unstable heart rhythm after its assessment, suspects an AMI, provides a time-sensitive intervention, or transports the patient urgently. Again, labeling such a case as ‘overtriage’ when the patient is assessed as clinically stable by paramedics completely ignores the initial risk of the patient presentation at dispatch.

**Fig. 1:**
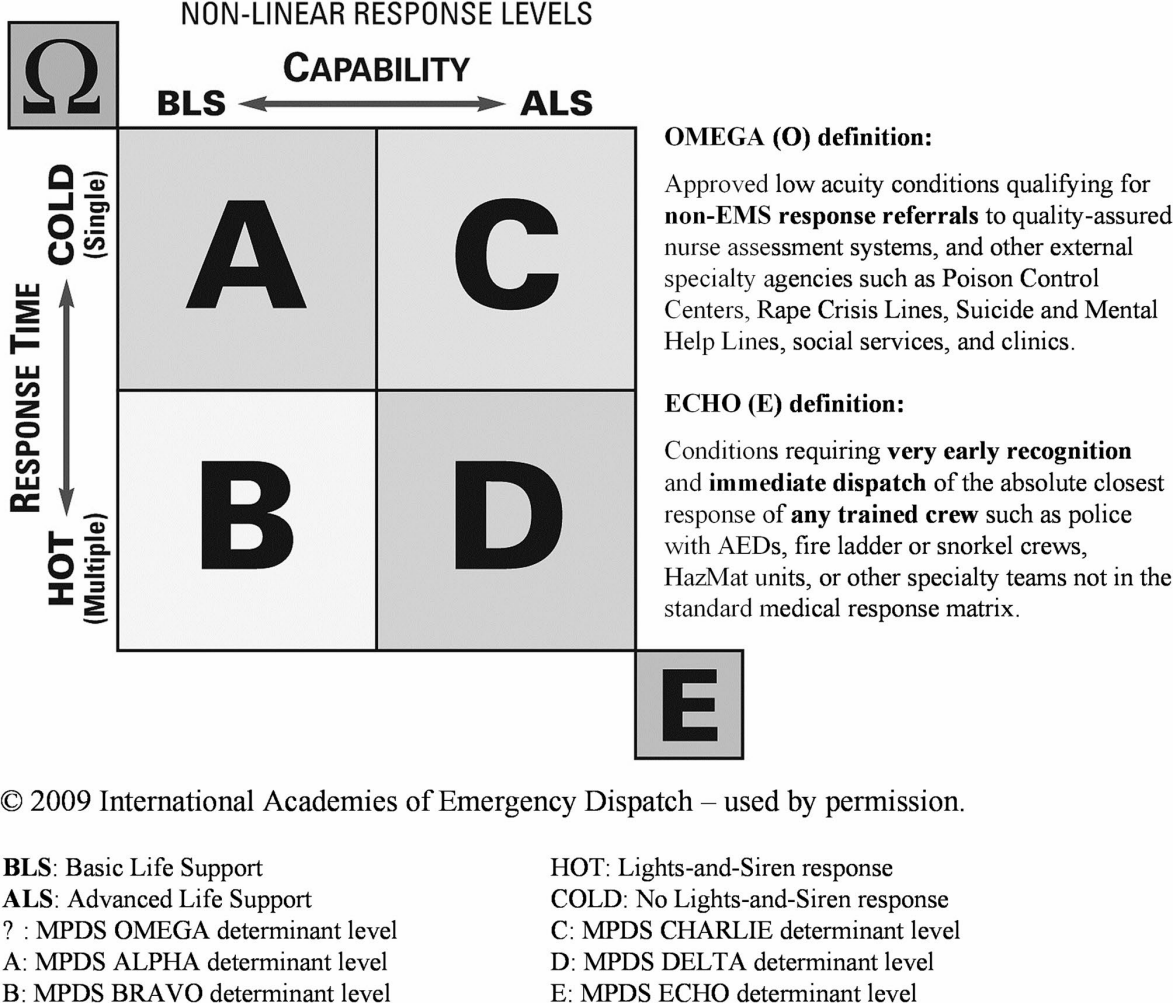
Medical priority dispatch system response matrix

In many ways the emergency communications dispatch center can be thought of as the entrance to the hospital emergency room, where a triage nurse makes a front-line decision as to which patients get seen immediately by the on-duty physician, which ones get put into an exam room where a nurse starts an assessment by taking vital signs and interviewing the patient, or which patients will sit in the waiting room until space and personnel are available for them. In that setting, it’s well understood that the triage nurse doesn’t simply take a quick look at each patient—and without physician assessment— immediately picks the few most serious patients to be admitted to the ICU, the CCU or another unit, then abruptly tells everyone else in line to go home because it’s been decided they’re not sick enough to be there. Likewise, the EMD is not equipped to engage in unsubstantiated and uncorroborated decisions when triaging patients over an emergency phone line. Comprehensive patient care is a process that requires a number of important, refined steps. In EMS, that starts with a well-trained, certified EMD, using an established protocol to assess and prioritize patient risk in a safe and consistent manner.

While the MPDS priority levels and Determinant Codes certainly have a strong association with patient acuity in the aggregate—i.e., the higher the priority of the code, the more likely the patient is to have a serious condition determined by the responding EMS crews [2–8]—they are designed, by necessity, to find any and all potentially serious patient conditions at the point of dispatch, so that a timely and sufficient EMS response and their needed level of evaluation can be assigned.

To be most effective, each MPDS user-agency must carefully assess its system resources and decide which of these resources are the best fit for each of the hundreds of MPDS Determinant Codes available to it. In doing so, it is not correct simply to lump priority levels CHARLIE, DELTA, and ECHO together, labeling them all as ‘urgent’ responses, and study them all as a single cohort, then draw conclusion about the entire MPDS coding system and individual Determinant Codes—all of which require careful consideration individually when assigning a user-defined response [9]. For example, consider an 82-year-old female who has had a ground-level fall with a possible hip fracture, awake and alert, no bleeding. Such a patient can be assigned one of three separate ALPHA-level Determinant Codes depending on the time the patient has been on the ground; 17-A-3 (Non-recent [≥ 6 h] injuries [without priority symptoms]); 17-A-3K (On the floor/ground for 1–2 h.), and 17-A-3M (On the floor/ground for > 2 h.). Each of these codes should be assigned a specific urgency and locally determined ‘P’ level in the City of Quebec system based on the risk—in this case the increasing risk of compartment syndrome when a patient is on the ground for an extended period.

Based on the information provided in this study, the best approach to conserving resources in its apparently strained system would be for the City of Quebec to re-examine its response assignment plan and seriously consider having more than five types of response to the hundreds of options the MPDS offers with its refined and well validated coding system and not simply “lumping” disparate levels (entire groups of codes) together.

## Data Availability

No datasets were generated or analysed during the current study.
